# High-Throughput Spectroscopy of Geometry-Tunable Arrays of Axial InGaAs Nanowire Heterostructures with Twin-Induced Carrier Confinement

**DOI:** 10.1021/acs.nanolett.4c04852

**Published:** 2024-11-04

**Authors:** Hyowon W. Jeong, Stephen A. Church, Markus Döblinger, Akhil Ajay, Benjamin Haubmann, Nikesh Patel, Jonathan J. Finley, Patrick W. Parkinson, Gregor Koblmüller

**Affiliations:** † Walter Schottky Institute, TUM School of Natural Sciences, Technical University of Munich, 85748 Garching bei München, Germany; ‡ Department of Physics and Astronomy and Photon Science Institute, The University of Manchester, Manchester M13 9PL, United Kingdom; § Department of Chemistry and Center for NanoScience, Ludwig-Maximilians-Universität München, 81377 Munich, Germany

**Keywords:** nanowire heterostructure, InGaAs, high-throughput spectroscopy, photoluminescence, transmission electron microscopy, rotational twin defects

## Abstract

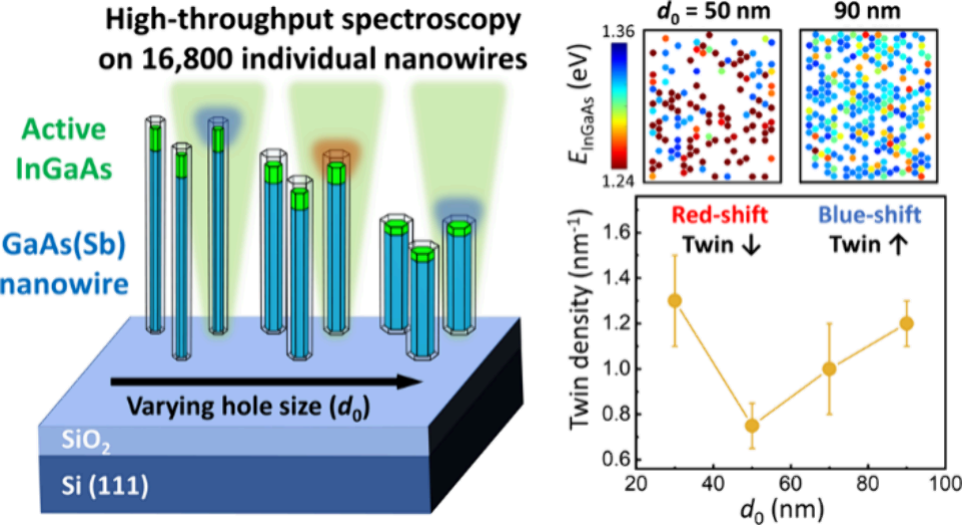

Predicting the optical properties of large-scale ensembles of luminescent nanowire arrays that host active quantum heterostructures is of paramount interest for on-chip integrated photonic and quantum photonic devices. However, this has remained challenging due to the vast geometrical parameter space and variations at the single object level. Here, we demonstrate high-throughput spectroscopy on 16800 individual InGaAs quantum heterostructures grown by site-selective epitaxy on silicon, with varying geometrical parameters to assess uniformity/yield in luminescence efficiency, and emission energy trends. The luminescence uniformity/yield enhances significantly at prepatterned array mask opening diameters (*d*_0_) greater than 50 nm. Additionally, the emission energy exhibits anomalous behavior with respect to *d*_0_, which is notably attributed to rotational twinning within the InGaAs region, inducing significant energy shifts due to quantum confinement effects. These findings provide useful insights for mapping and optimizing the interdependencies between geometrical parameters and electronic/optical properties of widely tunable sets of quantum nanowire heterostructures.

Free-standing III–V semiconductor nanowires (NWs) have emerged as a promising platform for a wide range of photonic and optoelectronic applications due to their unique material properties and versatile architecture.^1^ These one-dimensional (1D) nanostructures, with their high aspect ratios and ability to laterally relax strain within the NW, enable the effective formation of axial heterostructures,^2^ which are critical for realizing advanced devices such as quantum light sources,^3−9^ nanolasers,^10,11^ resonant tunneling diodes,^12,13^ and high-efficiency photodetectors.^14,15^ The ability to engineer such axial quantum heterostructures with precise control over composition, interface quality, size, and quantum confinement is essential for optimizing device performance and exploring new functionalities.

In particular, over the past decade, advances in noncatalytic, vapor–solid growth methods using selective area epitaxy (SAE) have opened new avenues for the synthesis of high-quality axial NW heterostructures with uniform, nontapered morphologies and well-defined heterointerfaces, suitable for various on-chip photonic applications.^16−23^ However, despite these advances, a significant gap remains in the understanding of how tunable array parameters, such as the pitch (interwire spacing) and mask-opening sizes in SAE templates, affect the optical emission properties of embedded quantum structures in NWs. Addressing this gap is crucial for optimizing the uniformity and yield of deterministic quantum light sources across large-scale arrays for a broad range of photonic and quantum photonic applications–especially in technologies where large numbers of identical quantum states (identical photons) are required as in e.g. on-chip Boson sampling devices for quantum computing.^24^ This requires intensive statistical studies employing high-throughput approaches that can systematically evaluate the luminescence properties of individual NWs within large arrays.^25^

In this work, we present geometry-tunable, on-chip arrays of optically active InGaAs axial NW heterostructures on silicon (Si), where the sensitivity of key optical properties on geometrical and structural parameters are revealed for the first time. The resulting heterostructures are confirmed through scanning transmission electron microscopy (STEM) and associated energy-dispersive X-ray spectroscopy (EDXS) on individual NWs. To statistically characterize the optical emission properties across a vast number of arrays, we perform high-throughput microphotoluminescence (μPL) spectroscopy on 16800 individual InGaAs NW heterostructures as a function of tunable SAE geometry parameters. We observe that not only the luminescence yield but also the emission energy does not follow a monotonic trend under varying parameters. We find that these trends cannot be accounted for by variations in the In composition, but rather suggest that other factors, such as microstructural features, play a more significant role. Specifically, we explore the impact of rotational twin defects on transition energy, utilizing experimental analysis including high-resolution (HR-) and high-angle annular dark-field (HAADF)-STEM, along with 1D numerical simulations, to elucidate twin-induced quantum confinement effects.

Figure 1a displays a schematic illustration of the axial NW heterostructures grown on a SiO_2_-masked Si(111) substrate with SAE prepatterns. As described in the Methods (S1, Supporting Information), the growth of the NW heterostructures is performed by an entirely catalyst-free process for all the respective materials, i.e., GaAs(Sb), InGaAs, and AlGaAs/GaAs, which build up the structure. Figure 1b,c shows a TEM micrograph of a representative single NW grown within a given array-field (mask-opening *d*_0_ = 30 nm, pitch *p* = 2 μm) along with an associated HAADF-STEM image (Figure 1c, top-left) as well as EDXS maps (blue = Ga, red = Al, green = In) scanned in the top region of the NW, respectively. Both the structural and compositional characterization confirm the successful insertion of the intended InGaAs segment (max. [In] ≈ 17%) along the NW axis with no radial In-deposition observed. The data also evidence the termination of the InGaAs segment by the subsequent capping with passivating Al_0.3_Ga_0.7_As/GaAs shells. For further quantitative compositional details, see Figure S2 in the Supporting Information. To conduct statistical investigations on geometry-dependent optical emission properties of the optically active InGaAs regions using high-throughput approaches, 42 fields with varying mask-opening sizes (*d*_0_ = 10–160 nm) and pitches (*p* = 2–10 μm) were fabricated, yielding a total of 16800 NWs. Figure 1d presents scanning electron microscopy (SEM) images of the as-grown NW arrays, taken at a 45° birds-eye view, on fields with a fixed pitch of *p* = 2 μm and varying mask-opening sizes of *d*_0_ = 30 (i), 50 (ii), and 90 nm (iii), respectively, as examples. An inverse relationship between NW lengths and diameters under varying *d*_0_ indicates typical characteristics of the noncatalytic growth mechanism.^26−30^ Similar trend is also observed in the dimensions of the InGaAs segments, in agreement with previous work,^23^ and as confirmed by associated STEM-EDXS data in the Supporting Information (Figures S3 and S4). Note that irrespective of the mask-opening size, *d*_0_, the radial and axial dimensions of the InGaAs segments are well above the size range for which quantum effects occur in low-[In] InGaAs.^31,32^

**Figure 1:**
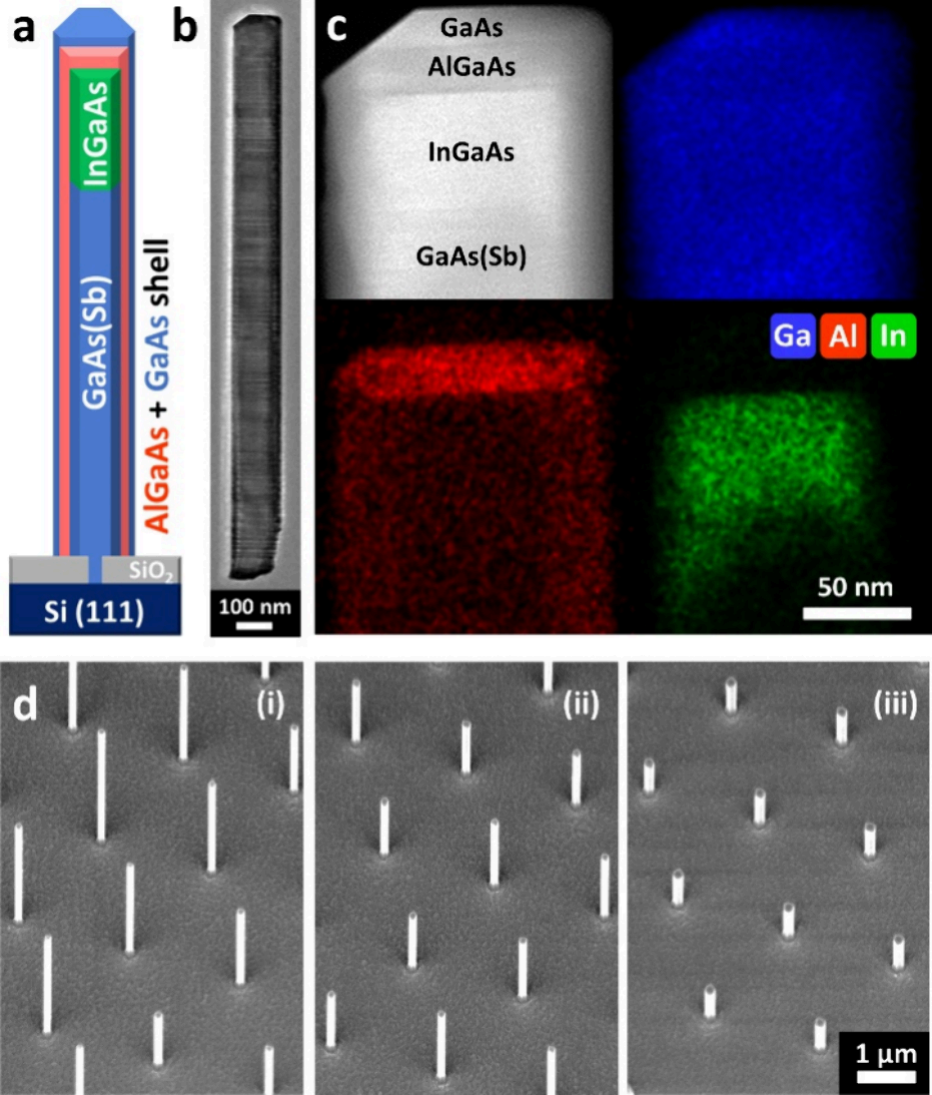
Structural and compositional properties of NW heterostructures. (a) Schematic illustration of the NW heterostructures grown site-selectively on a prepatterned SiO_2_/Si(111) substrate. (b) TEM micrograph of a NW grown with *d*_0_ = 30 nm. (c) Associated HAADF-STEM micrograph (top-left) and EDXS elemental maps of Ga (blue), Al (red), and In compositions (green), respectively. (d) SEM images of NW arrays with a fixed pitch of *p* = 2 μm and varying *d*_0_ = 30 (i), 50 (ii), and 90 nm (iii), respectively.

The optical quality of the embedded InGaAs segment in a single as-grown NW was assessed using room temperature μPL, employing an experimental setup adapted from previous works,^25^ which is discussed further in the Methods section (S1, Supporting Information). Figure 2a shows a representative spectrum measured from a NW with an InGaAs active region, which has been fit using a model for band-to-band recombination (details in S1, Methods, Supporting Information) for quantitative analysis of the spectral properties. Two emission peaks are observed with peak energies (and wavelengths) of ≈1.41 eV (882 nm) and ≈1.31 eV (944 nm), corresponding to bandgap energies of 1.38 and 1.29 eV, respectively. However, it must be noted that due to unquantified effects, such as strain, it is challenging to accurately assess the composition from these PL data. Nevertheless, the higher energy peak can be attributed to carrier recombination in the GaAs(Sb) NW core, which contains a small amount of Sb (≈2–4%),^26,30^ and the lower energy peak to emission from the InGaAs segment, containing [In] ≈ 15–20%.^23^ The intensity of the InGaAs peak is ≈37% of the total spectral intensity, despite the segment comprising only ≈3% of the NW length. This suggests effective carrier recombination within the optically active InGaAs region, which likely acts as a carrier sink, facilitating efficient luminescence.

**Figure 2:**
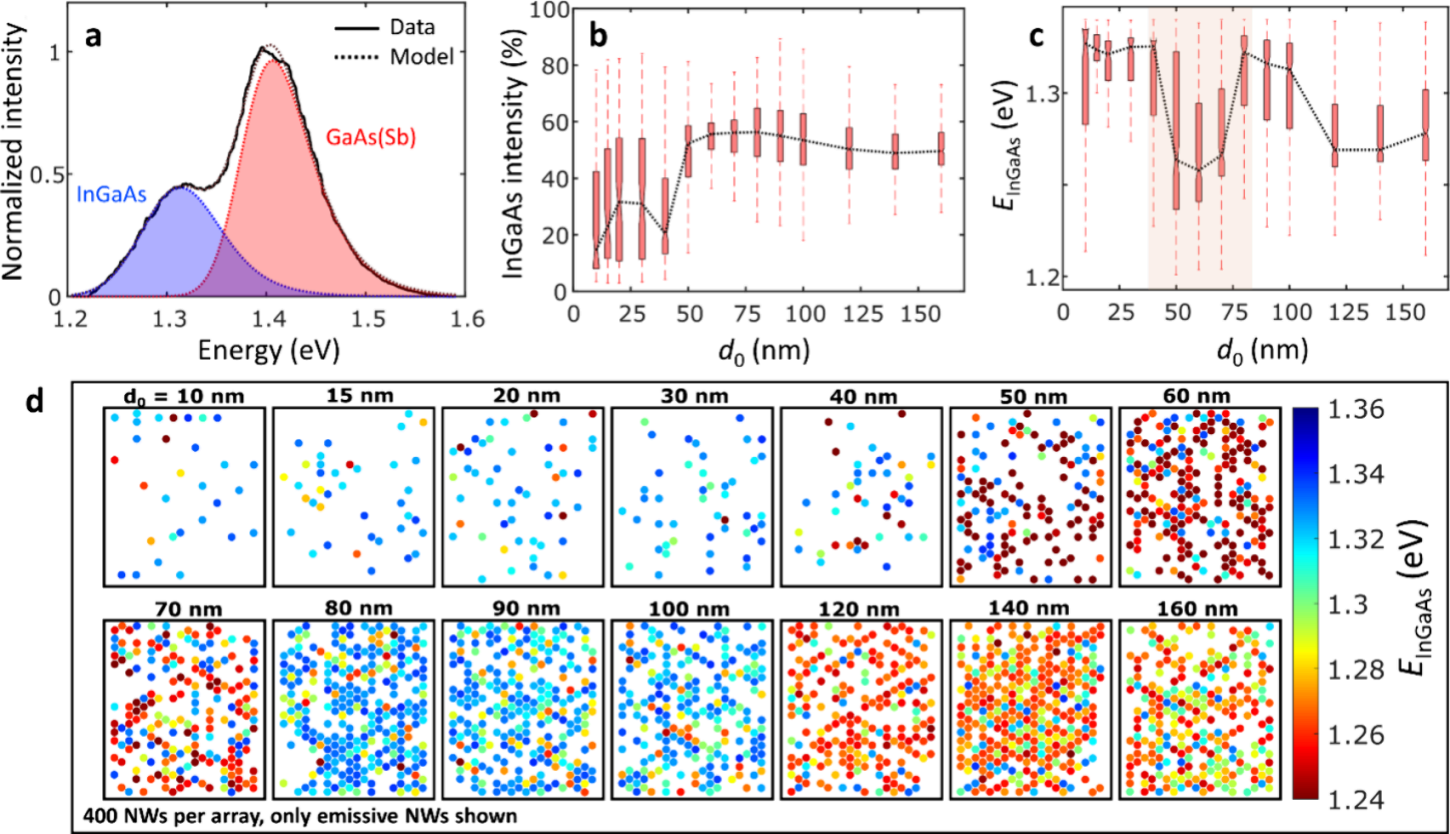
High-throughput PL results of NW arrays. (a) Room temperature PL spectrum measured for a single NW with an InGaAs active region. The spectrum is fit with a band-to-band recombination model (see S1, Methods, Supporting Information).^33^ (b) Statistical distribution of InGaAs PL intensity (normalized to the total emission intensity) from 5600 individual NWs in arrays with *p* = 10 μm, plotted as a function of *d*_0_ ranging from 10 to 160 nm. (c) Distribution of InGaAs bandgap energies (*E*_InGaAs_) for each array, plotted as a function of *d*_0_. The dotted line connects the median values of each array and serves as a guide to the eye. (d) Map of the bandgap of the InGaAs segment in 5600 individual NWs, with *p* = 10 μm and *d*_0_ ranging from 10 to 160 nm. Each colored data point (dot) corresponds to a NW that emits at a specific energy, with its bandgap given by the respective color-axis scale. Each array contains 400 NWs, and only those with observed InGaAs emission are shown, while NWs with no InGaAs emission are marked invisible.

The spectral width, characterized by the disorder parameter (σ, see S1, Methods, Supporting Information), provides an assessment of the uniformity of each NW region. The σ values for the GaAs(Sb) and InGaAs peaks are 24 and 38 meV, respectively. The GaAs(Sb) disorder parameter is relatively large compared to VLS-grown GaAs NW cores, which typically exhibit values of ≈10 meV,^33^ possibly due to compositional fluctuations and strain in the NW. On the other hand, the InGaAs disorder (likely due to compositional and size fluctuations of the layers) is comparable to values obtained for core/shell quantum well heterostructures,^33^ suggesting that the InGaAs segment has optical quality similar to more established core/shell structures.

Figure 2b–d demonstrates the above measurement and fitting process repeated for 5600 individual NWs with *p* = 10 μm using our high-throughput μPL setup.^25^ The results from a further 11200 NWs, produced during the same SAE growth, with pitches of 5 and 2 μm, are provided in Figure S5 in the Supporting Information, demonstrating the same trends as those shown here. This approach enables a robust determination of the impact that the SiO_2_-mask opening properties (i.e., *d*_0_ and *p*) have on the InGaAs luminescence, as well as an assessment of the repeatability and reliability of the growth process–crucial factors when scaling up these devices for various applications. Figure 2d shows the bandgap of the InGaAs segment mapped for the same 5600 individual NWs with *p* = 10 μm, as an example. 400 individual NWs were measured for each value of *d*_0_: the majority (90–100%) of these NWs exhibit the GaAs(Sb) emission peak, however a reduced number of NWs have distinct InGaAs emission. NWs with no such distinct InGaAs emission are marked as invisible on the maps. Remarkably, there are two distinct regimes observed in the data set. For hole diameters greater than 50 nm, more than 88% of NWs show emission from InGaAs, thus demonstrating reliable formation of the InGaAs active region. In this regime, the luminescence intensity from the InGaAs segments is insensitive to changes in *d*_0_. This is shown in Figure 2b, where the intensity of each InGaAs segment has been normalized to the total intensity from each NW. This normalization is required to minimize the impact of the pumping and light extraction efficiency, which vary with optical alignment on each NW and have a significant impact on the collected light levels. The median intensity is around 55% and there is an interquartile range (IQR) of less than 20% for all NW arrays. This indicates that the relative recombination efficiency in the segment and in the GaAs(Sb) stem is homogeneous across the population.

In contrast, for hole diameters below 50 nm, the emission from the NWs is impacted. As shown in Figure 2d, the percentage of NWs exhibiting an InGaAs emission peak drops to 25%. It is important to note that this does not preclude the presence of InGaAs in these NWs, only that the intensity of the InGaAs emission has dropped below the detection sensitivity limit. This may result from reduced NW diameters in these arrays, leading to a smaller volume of emissive material and reduced absorption of the laser excitation. Simultaneously, Figure 2b shows that for NWs where distinct InGaAs emission is detectable, the median intensity of the InGaAs peak drops to as low as 15%, and the IQR increases to as high as 43%. This may indicate either a reduced recombination efficiency in the segment, an increased recombination efficiency in the GaAs(Sb) stem, or a lower carrier density relative to the rest of the NW, along with more variability in the segment across the population. Therefore, SiO_2_-mask opening diameters above 50 nm are significantly advantageous for achieving optimal uniformity and InGaAs emission performance.

Furthermore, while the map in Figure 2d presents the InGaAs bandgap energy of individual NWs scaled between 1.24 eV (red) and 1.36 eV (blue), Figure 2c shows its quantitative evolution as a function of *d*_0_. Interestingly, these high-throughput data reveal an anomalous relationship between *d*_0_ and InGaAs bandgap energy (e.g., marked with a red box). The median bandgap is ≈1.32 eV for *d*_0_ less than 50 nm, and this decreases to ≈1.26 eV for *d*_0_ values between 50 and 70 nm. Similarly, while the median bandgap increases to 1.31 eV for *d*_0_ between 80 and 100 nm, it reduces again to 1.27 eV for larger *d*_0_ values. This behavior is consistently observed in ensembles from other equivalent NW array samples when analyzed using low-temperature (10 K) macro-PL spectroscopy (see Figure S6, Supporting Information). Such nonmonotonic trends cannot be explained by variations in the In-content [In] of the InGaAs segments, which remains almost constant across the investigated NWs, taking experimental errors in EDXS detection into account (Figure S4, Supporting Information). In fact, some of the results indicate even the opposite values, with slightly lower [In] observed in an InGaAs NW sample with a red-shifted bandgap, which is counterintuitive, thus ruling out variations in InGaAs composition as the key cause. Likewise, the size dimensions of the InGaAs segments are fairly large (>100 nm radially, and >25 nm axially), such that quantum confinement effects can be also neglected.

Hence, other factors must be responsible for the observed nonmonotonic trends, such as, for example, the crystal structure within the given sets of NW-arrays. Particularly, we anticipate that microstructural properties, specifically rotational twin defects, play a substantial role in charge carrier recombination and resulting transition energy. Indeed, recent studies have demonstrated that variations in array-geometry parameters (such as pitch) result in different densities of twin defects in GaAs NW-arrays, affecting their optical transition energies.^34^Figure 3a (top) shows a HR-HAADF-STEM micrograph of a typical InGaAs section that contains rotational twin domains (separated by orange dotted lines) formed axially along the (111)B direction. As schematically illustrated in Figure 3a (bottom), the formation of a rotational twin can be considered an inclusion of a wurtzite (WZ) monolayer within the zinc-blende (ZB) domain matrix, resulting in a type-II band alignment at the interfaces. Consequently, holes are localized in the valence band (VB) of the WZ monolayer region (twin defect) along the NW growth direction, while electrons remain in the conduction band (CB) of the ZB domains, forming spatially indirect excitons.^26,30,35−38^Figure 3b presents three representative HR-HAADF-STEM micrographs of twin layers, marked by arrows within an arbitrary 10 nm long InGaAs segment region along the growth axis, obtained from different hole opening sizes of *d*_0_ = 30 (i), 50 (ii), and 90 nm (iii), respectively. Figure 3c displays the mean twin defect densities analyzed in multiple NW samples with *d*_0_ = 30, 50, 70, and 90 nm. In total 2–3 NWs/sample were probed, which proved sufficient to verify the observed trend, thanks to the high-uniformity selective-area growth process (cf. Figure 1d). This quantitative trend (illustrated with a red box) exhibits a significant decrease in twin density at *d*_0_ = 50 nm, marking its low point, followed by an increase toward *d*_0_ = 70 and 90 nm. Such trend evidences a distinct correlation with the nonmonotonic luminescence properties discussed in Figure 2 (and in Figures S5 and S6, Supporting Information), corresponding to the red- and blue-shifts in emission energy, respectively.

**Figure 3:**
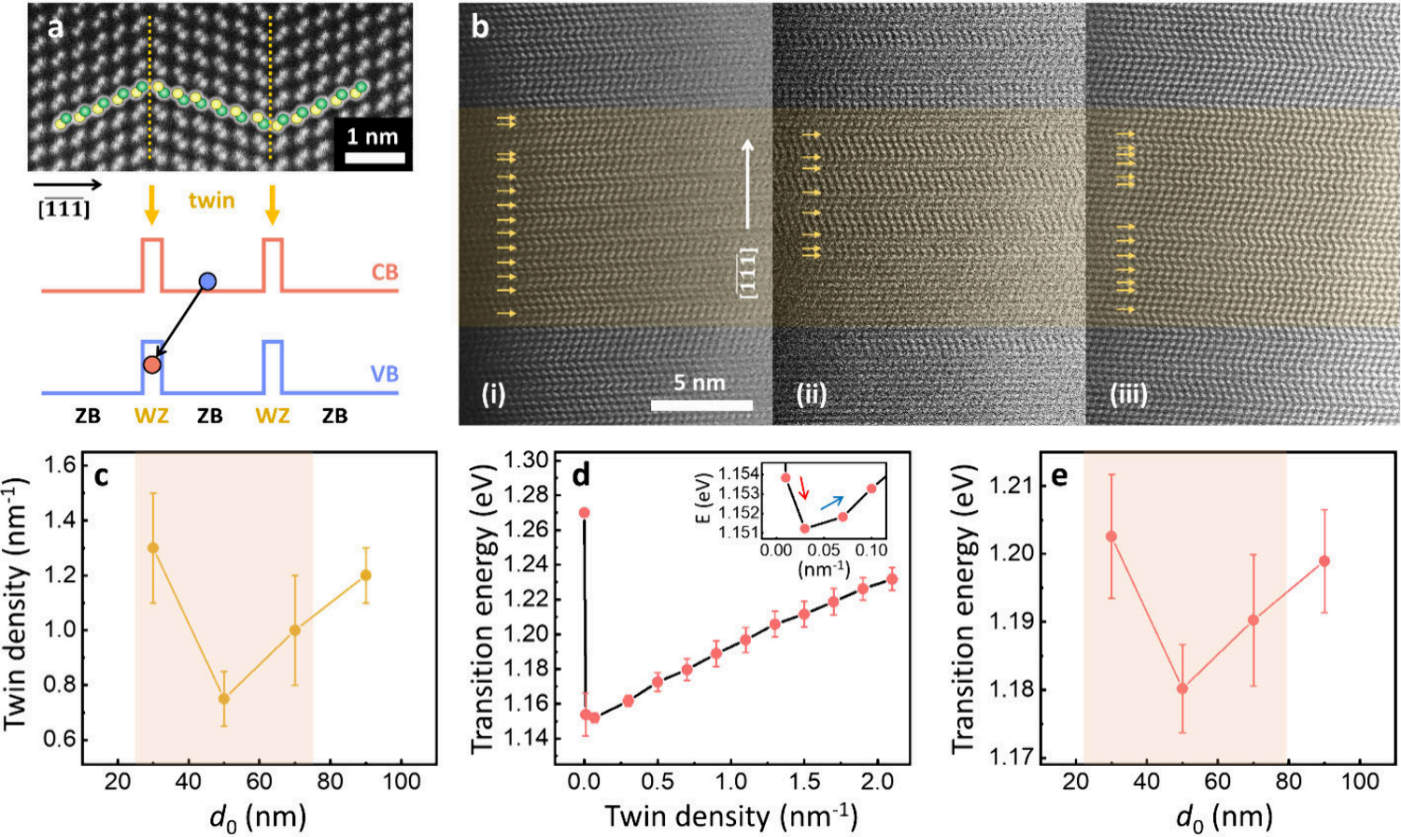
Correlation of rotational twin defect and transition energy. (a) HR-HAADF-STEM micrograph of rotational twins (orange dotted lines) formed axially along the (111)B direction in the ZB domains of an InGaAs NW (top), and schematic illustration of the type-II band lineups due to twins–insertion of WZ monolayers (bottom). (b) Representative HR-HAADF-STEM micrographs of the InGaAs segments, obtained from growth fields with varying *d*_0_ = 30 (i), 50 (ii), and 90 nm (iii), respectively. Individual twins are marked by arrows in an arbitrary 10 nm InGaAs region (orange box). (c) Quantitative trend of mean twin density in the InGaAs segments with respect to *d*_0_. (d) 1D simulation of transition energy in an InGaAs segment of [In] = 17% as a function of twin defect density ranging from 0 to 2.1 nm^–1^ (inset: magnified graph at the low twin densities from 0.01 to 0.1 nm^–1^; red and blue arrows indicate red- and blue-shifts in transition energy, respectively), and (e) as a function of *d*_0_ in accordance with the input twin densities from (c).

To support the correlation between twin defect density and observed emission energy, 1-dimensional (1D) numerical simulations were conducted using nextnano++, where the transition energy between electron and hole ground states at 10 K was calculated by solving the Schrödinger equation. With the input In-content kept constant at 17%, the InGaAs segment was modeled as a randomized sequence of 500 layers, representing either the ZB or WZ phase, to reflect the statistical formation of twins (details in S1, Methods, Supporting Information). Figure 3d shows the simulation results of transition energy as a function of twin defect density varying between 0 and 2.1 nm^–1^. Notably, within the lowest twin densities up to 0.03 nm^–1^ (magnified view from 0.01 to 0.1 nm^–1^ in the inset), an abrupt red-shift in transition energy of ≈120 meV occurs, which is attributed to the emergence of WZ segments and the increasing incidence of type-II indirect recombination at a lower energy level than direct excitons.^35,36^ Conversely, as twin density increases further from 0.03 up to 2.1 nm^–1^, the trend shifts from red- to a significant blue-shift of up to ≈80 meV. This suggests that a substantial increase in twin density may induce quantum confinement effects for the indirect excitons, leading to a blue-shift in transition energy due to the reduction in spatial extent of the electron and hole wave functions.^26,30,37,38^Figure 3e presents the simulation results as a function of *d*_0_, according to the input twin densities from Figure 3c, which clearly demonstrates a similar nonmonotonic trend in transition energy as shown by the experimental data in Figure 2. While the calculated transition energies are offset to lower energies compared to the data in Figure 2, an even better agreement is seen by the data shown in Figure S6 (Supporting Information). This is most likely due to the different excitation conditions, i.e., much lower excitation power density, used in this case. Overall, these findings indicate that, while the exact mechanism by which varying mask opening sizes lead to nonmonotonic changes in twin defect density remains unknown to date, the twin-induced charge carrier confinement plays a substantial role in tuning the emission energy in this axial NW heterostructure. This finding will have important implications on the emission optimization on future axial NW-heterostructure based quantum light sources.

In summary, we explored the geometry-dependent luminescence properties of optically active InGaAs axial NW heterostructures, grown by selective area epitaxy in arrays with large geometrical parameter spaces. Employing high-throughput μPL spectroscopy, which enabled the analysis of 16800 individual NWs, we observed distinct emissions from the InGaAs active region and assessed their uniformity and yield, which significantly increased at *d*_0_ greater than 50 nm. Furthermore, the InGaAs emissions revealed a nonmonotonic trend in transition energy with varying SiO_2_-mask opening sizes, which cannot be attributed to compositional differences. Investigation of the microstructural features along the InGaAs regions, combined with correlated 1D numerical simulations, demonstrated that an increase in twin defect density leads to a substantial blue-shift in transition energy, highlighting the significant role of twin-induced quantum confinement effects in shaping the emission characteristics of the NW heterostructures. The insights gained from these high-throughput studies and the related understanding of twin-induced emission characteristics will enable further fine-tuning of NW properties, and especially tailoring the emission of axial NW quantum heterostructures for next-generation quantum light sources in on-chip quantum photonic integrated circuits.

## Supporting Information Available

Methods, including selective-area molecular beam epitaxy, high-throughput μPL spectroscopy, structural analysis, and numerical simulation; STEM-EDXS quantitative analysis; EDXS maps for varying *d*_0_; EDXS quantitative analysis for varying *d*_0_; High-throughput PL data for different pitches; Macro-PL spectroscopy for varying array geometry parameters (PDF)
