# Supplementary material for: High-Throughput Spectroscopy of Geometry-Tunable Arrays of Axial InGaAs Nanowire Heterostructures with Twin-Induced Carrier Confinement
Source: Nano Lett. 2024 Nov 4;24(45):14515–21. doi: 10.1021/acs.nanolett.4c04852 (PMC11565757; doi:10.1021/acs.nanolett.4c04852)
Supplement: Supplementary file 1 — nl4c04852_si_001.pdf [file nl4c04852_si_001.pdf]

Supporting Information for

High-Throughput Spectroscopy of Geometry-Tunable Arrays of Axial InGaAs Nanowire Heterostructures with Twin-Induced Carrier Confinement

Hyowon W. Jeong[1,], Stephen A. Church[2,*], Markus Döblinger[3], Akhil Ajay[1], Benjamin Haubmann[1], Nikesh Patel[2], Jonathan J. Finley[1], Patrick W. Parkinson[2], Gregor Koblmüller[1,*]*

[1]Walter Schottky Institute, TUM School of Natural Sciences, Technical University of Munich, 85748 Garching bei München, Germany

[2]Department of Physics and Astronomy and Photon Science Institute, The University of Manchester, Manchester M13 9PL, United Kingdom

[3]Department of Chemistry and Center for NanoScience, Ludwig-Maximilians-Universität München, 81377 Munich, Germany

*Corresponding authors.

E-mail: Hyowon.Jeong@wsi.tum.de (H.W.J.); stephen.church@manchester.ac.uk (S.A.C.); Gregor.Koblmueller@wsi.tum.de (G.K.)

S1. Methods

Selective-Area Molecular Beam Epitaxy (SAE)

For the growth of nanowires (NW), a solid-source Gen-II molecular beam epitaxy (MBE) system was used, which is equipped with conventional effusion cells for group-III elements (In, Ga, Al) and Veeco valved cracker cells for group-V elements (As, Sb). The As species were supplied as uncracked As_4, and the Sb species as Sb_2 molecules. For fabricating nanopatterns as SAE templates, commercial single-side polished 2-inch p-type Si (111) wafers, covered by a thermally grown ≈20 nm-thick SiO_2 mask layer, were used as substrates. Employing electron beam lithography (EBL), reactive ion etching (RIE), and wet chemical etching (buffered hydrofluoric (HF) acid), patterns of periodic hole opening arrays were written on the SiO_2 mask layer with varying nominal circular diameters (d_0 = 10–160 nm) and pitches (p = 2–10 nm). Using the prepatterned hole arrays, catalyst-free NWs were grown by the growth procedures reported in our earlier work.[1-4] First, GaAs(Sb) NWs were grown using Ga flux of 0.35 Å/s, As-BEP (beam equivalent pressure) of 5.5×10^{-5} mbar, Sb-BEP of 3×10^{-7} mbar, respectively, for a fixed growth time of 60 min at a substrate temperature of 630°C. Such non-catalytic GaAs(Sb) NWs containing only a small Sb molar fraction (≈2–3%) serve as a stem with improved morphological, microstructural, and optical properties due to so-called Sb-surfactant effect.[3, 4] For the axial insertion of InGaAs segments on top of the GaAsSb core NWs, the substrate temperature was ramped down to 590 °C and In-flux of 0.30 Å/s was applied, while keeping the Ga-flux of 0.35 Å/s and As-BEP of 5.5×10^{-5} mbar fixed.[5] Additionally, at a further cooled substrate temperature of 500 °C, a thin $Al_{0.3}Ga_{0.7}As$ passivation layer was radially grown (≈5 nm) to prevent non-radiative surface recombination, followed by a subsequent GaAs capping (≈3 nm) to protect the Al-containing layer from oxidation.[6]

High-throughput micro-photoluminescence (μPL) spectroscopy

To determine the luminescence properties of individual NWs, and to assess the uniformity and yield of the InGaAs segment growth across a full mask-set, the sample was investigated using a high-throughput confocal μPL setup. Each as-grown NW was excited at room temperature using a continuous wave laser with a wavelength of 532 nm, an excitation spot diameter of 1 μm and a power density of 1 kW/cm^2. The emission from a 5 μm diameter area centered on the NW was collected using a 20x microscope objective lens and directed through a 105 μm diameter, 0.22 NA optical fiber. The PL spectrum was measured using a Horiba iHR550 spectrometer with a 150 lines/mm grating and a SynapsePlus high speed CCD array, resulting in a spectral resolution of 1 nm. Additional details for this experimental setup can be found at the Ref. **[7]**. The measured PL spectra were corrected for the spectral response of the system by using a blackbody reference source. As shown in Figure 2 in the main part, each spectrum exhibits an emission peak from recombination in the GaAs(Sb) NW, and a lower-energy peak due to recombination in the InGaAs segment. Each peak was fit with a previously published model,**[8]** which considers the density of states and a Boltzmann occupation function for each recombination pathway:

$$B_{GaAs(Sb)}(E) = \sqrt{E - E_{GaAs(Sb)}}\,\exp\left(\frac{-\left(E - E_{GaAs(Sb)}\right)}{k_B T}\right)$$

$$B_{InGaAs}(E) = \exp\left(\frac{-(E - E_{InGaAs})}{k_B T}\right) \text{ for } E > E_{InGaAs}$$

where E is the photon energy, $E_{GaAs(Sb)}$ and E_{InGaAs} is the bandgap of the GaAs(Sb) and the InGaAs region, respectively, k_B is the Boltzmann constant and T is the carrier temperature (assumed to be 300 K). It is also assumed that the InGaAs segment has a 2D density of states and the rest of the NW has a 3D density of states, and that band-filling effects are negligible. To account for

inhomogeneity in each segment of the NW, these functions were convoluted with a Gaussian to finalize the model for intensity, I:

$$I(E) = A_{InGaAs}B_{InGaAs}(E) \times \exp\left(\frac{-E^2}{2\sigma_{InGaAs}^2}\right) + A_{GaAs(Sb)}B_{GaAs(Sb)}(E) \times \exp\left(\frac{-E^2}{2\sigma_{GaAs(Sb)}^2}\right)$$

where A are scaling factors, and σ is the disorder parameter, defined by the standard deviation of the Gaussian. This analysis was performed for a total of 16,800 NWs from the same growth batch.

Structural Analysis

To view the growth yield and morphology of the NW arrays, scanning electron microscopy (SEM) was employed using an NVision 40 FIB-SEM apparatus from Carl Zeiss. All images were recorded at 45° bird-eye view and tilt-corrected by the SEM software to show the actual lengths. To investigate the microstructural features of the NW heterostructures, high-resolution (HR-) and high-angle annular dark-field scanning transmission electron microscopy (HAADF-STEM) was performed along the InGaAs regions of the individual 2–3 NWs/sample, which were mechanically transferred from identical SAE array fields onto carbon-coated copper grids and probed in a FEI Titan Themis TEM operating at 300 kV. Additionally, energy-dispersive x-ray spectroscopy (EDXS) and associated mapping was performed to characterize the compositional profiles.

Numerical Simulation

To calculate the expected emission energy of an InGaAs segment with a certain nominal twin density at 10 K, the transition energy between the ground states of electrons and holes was simulated by numerically solving the Schrödinger equation using nextnano++. To account for the statistical nature of the formation of twins, the simulated InGaAs segment was generated as a randomized sequence of a total of 500 layers, which could be either in zinc-blende (ZB) or in

wurtzite (WZ) phase. A twin was hereby defined as the interface between a ZB and a WZ phase segment, and the formation probability of a twin per interface was

$$p = \frac{\rho \cdot a_{GaAs}}{\sqrt{3}}$$

with ρ the nominal twin density per nm and a_{GaAs} = 5.65 Å the lattice constant of GaAs.[9] For fixed ρ, the transition energy still depends on the concrete positions of the twins. This effect was eliminated by performing the simulation for 100 randomized layer arrangements per set of parameters and taking the mean values for the transition energy. Hereby, only deviations of up to 0.025 nm^{-1} of the actual from the nominal twin density were tolerated. To isolate the effect of twinning, the In-content was kept fixed at 17 % for these simulations, which is a reasonable assumption, since the In-contents measured by EDXS are prone to errors larger than the observed variations across different hole opening diameters. Also, strain was disregarded in the simulations. The electron- and hole wave function depends on the position of the conduction- and valence band, respectively, as well as the corresponding effective mass. For the simulations in this work, only the heavy-hole (HH) band was considered for holes. While the material parameters of InGaAs in ZB phase are already contained in the nextnano material database, they were manually defined for the WZ phase. This was done by defining WZ GaAs and WZ InAs separately and interpolating linearly with the In-content to obtain WZ InGaAs. A complete list of all relevant material parameters of WZ GaAs and InAs for solving the Schrödinger equation are provided in **Table 1**.

	$m_{e,WZ}$ (m_0)	$m_{h,WZ}$ (m_0)	ΔE_{HH} (eV) = $E_{HH,WZ} - E_{HH,ZB}$	$E_{g,WZ}$ (eV)
GaAs	1.092 [9]	0.766 [9]	0.122 [9]	1.547 [10]
InAs	0.037 [11]	0.9738 [11]	0.045 [12]	0.037 [11]

Table 1. Material parameters of WZ GaAs and InAs

S2. STEM-EDXS quantitative analysis

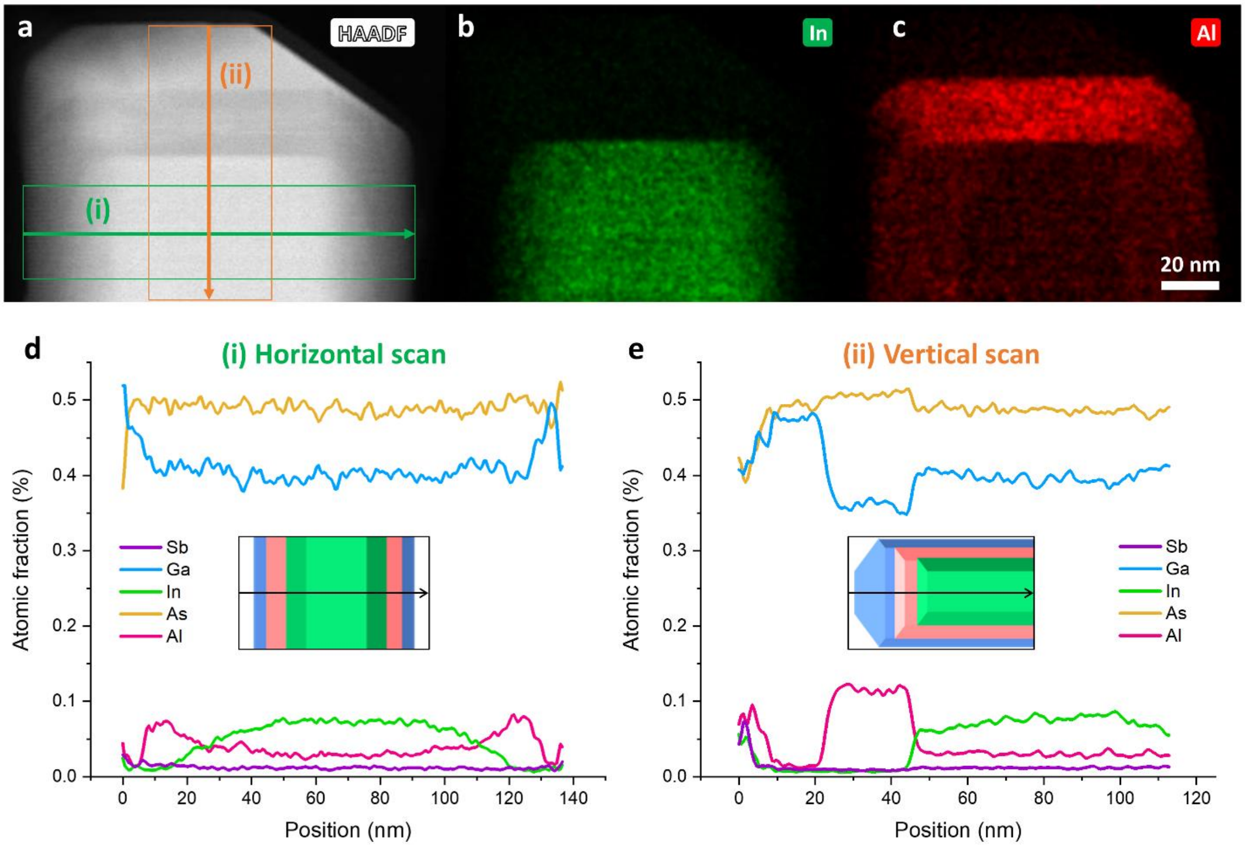

Figure S2: Compositional analysis of the InGaAs NW heterostructure. (a) HAADF-STEM micrograph and (b,c) associated EDXS elemental maps of In- (b, green) and Al compositions (c, red), recorded in the upper region of the NW heterostructure grown with d_0 = 30 nm. (d,e) EDXS atomic fraction profile line scanned horizontally (d) and vertically (e) across the In-containing segment (as indicated in (a) by green- (i) and orange arrow (ii), and also in the respective insets). The III-V alloy compositions (e.g., In-content; [In]) correspond to twice the respective atomic fractions in these plots. For example, the In-rich region contains a maximum [In] ≈17.4 % in this NW.

S3. EDXS maps for varying d_0

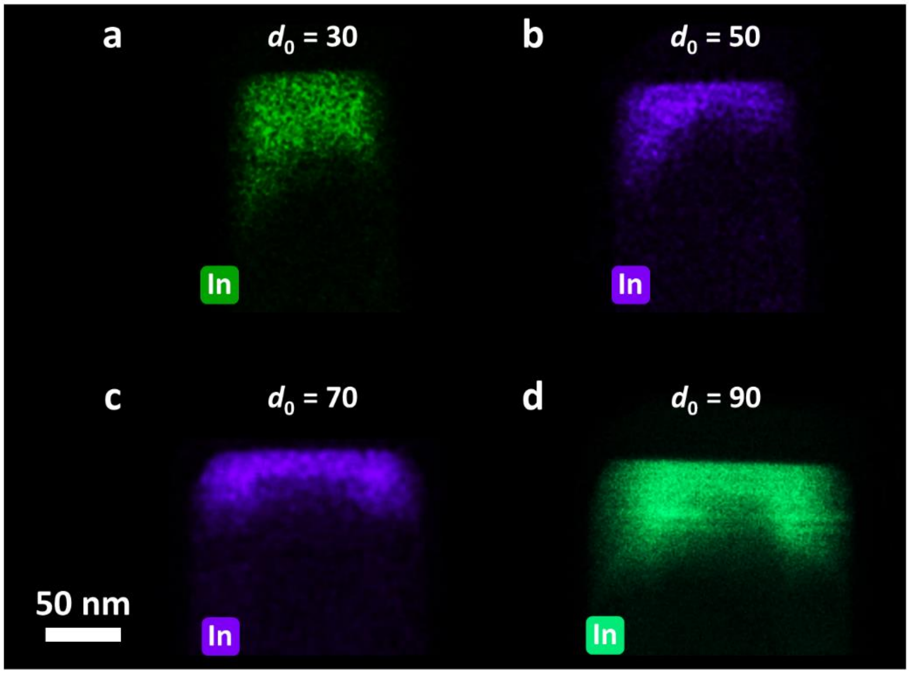

Figure S3: EDXS elemental distribution map of In recorded in the upper region of the NW heterostructure with varying mask-opening sizes of d_0 = 30 nm, (b) 50 nm, (c) 70 nm, and (d) 90 nm, respectively.

S4. EDXS quantitative analysis for varying d_0

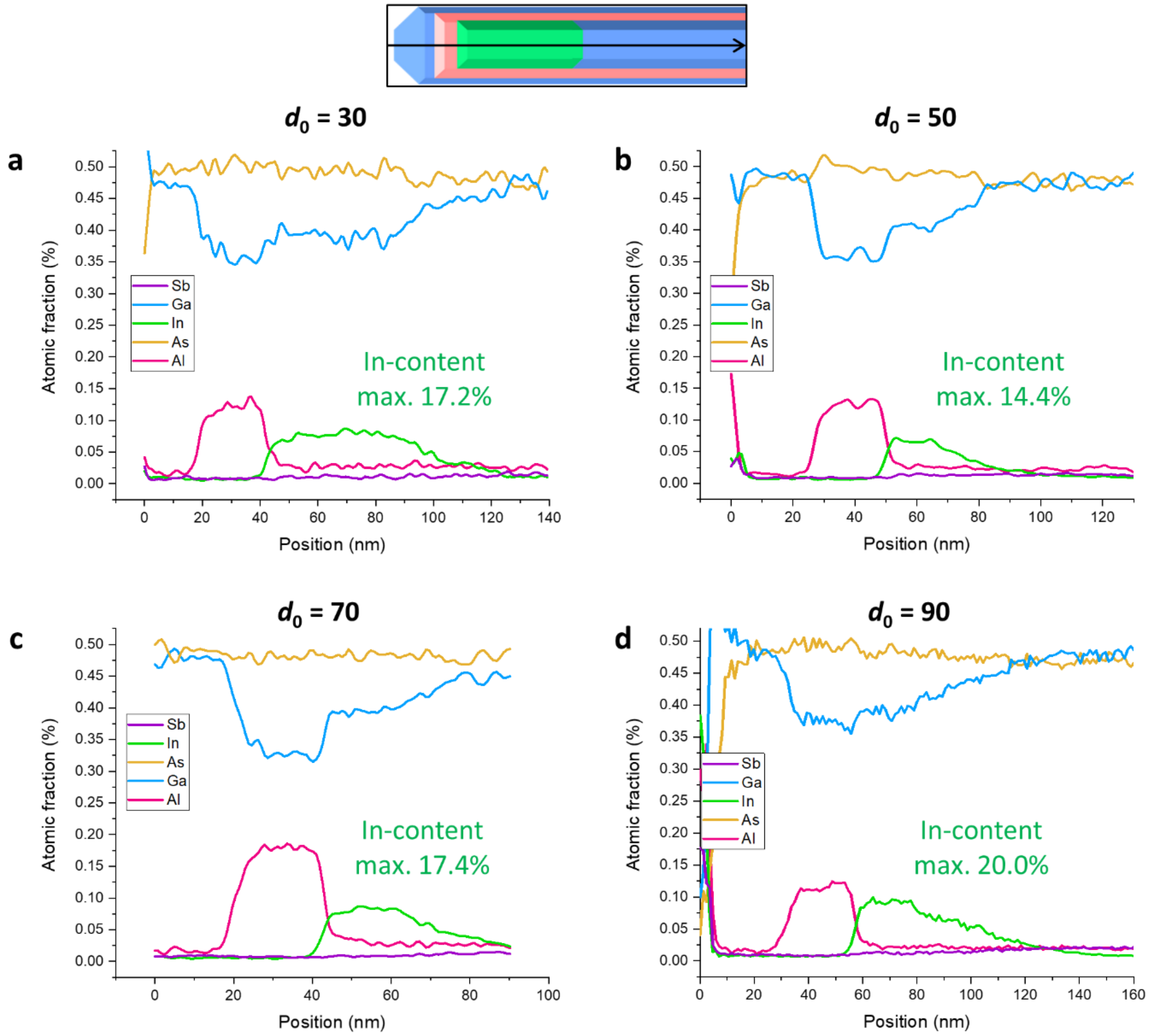

Figure S4: EDXS atomic fraction profile line scanned vertically across the In-containing segment for (a) d_0 = 30 nm, (b) 50 nm, (c) 70 nm, and (d) 90 nm, respectively. The alloy compositions (e.g., In-content; [In]) represent twice the respective atomic fractions for each group III or V element.

S5. High-throughput PL data for different pitches

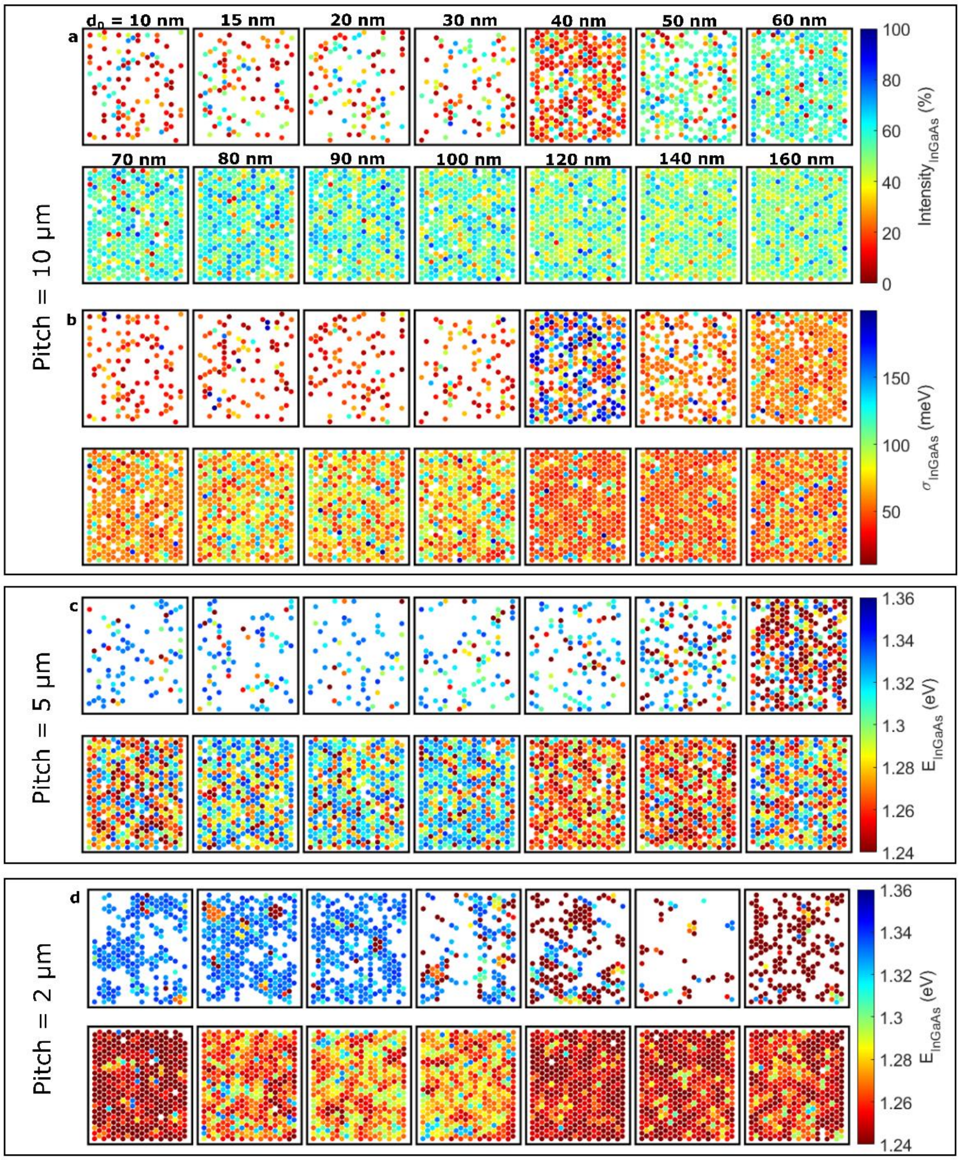

Figure S5: High-throughput PL data for different pitches. (a,b) Additional data for 10 μm pitch NWs: intensity of the InGaAs segment emission normalized to the total spectral intensity (a), and the disorder parameter (σ) for the InGaAs emission (b). (c,d) Bandgap energy of the InGaAs segment for 5 μm pitch (c) and 2 μm pitch NWs (d). The colored dots represent the bandgap of individual NWs. Each array contains 400 NWs, and only those with observed InGaAs emission are shown, while NWs with no InGaAs emission are marked invisible.

S6. Macro-PL spectroscopy for varying array geometry parameters

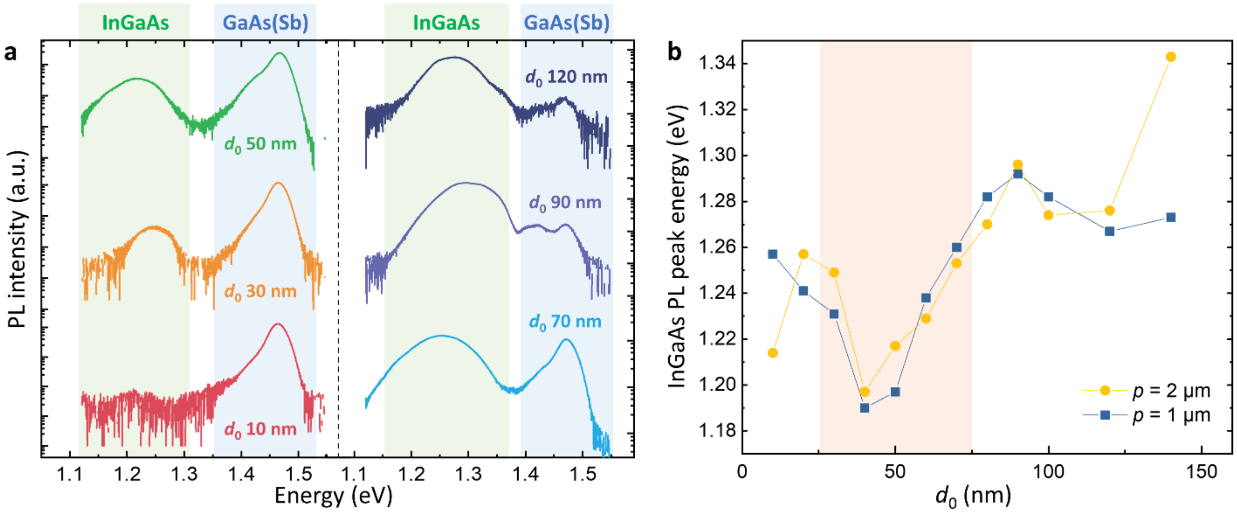

Figure S6: (a) Low-temperature (10 K) macro-PL spectra from as-grown GaAs(Sb)/InGaAs NW arrays with a fixed pitch of p = 2 μm and varying hole opening diameters (d_0) ranging from 10 nm to 120 nm. A mode-locked tunable Ti:sapphire laser (780 nm) with a repetition rate of 80 MHz and a pulsed width of ≈100 ps was used for excitation. The excitation power density (P_{exc}) was ≈0.2 μW/μm^2, with a spot size of ≈12.4 μm allowing for probing a large area of each NW array field. (b) Quantitative trends of the PL peak energy from InGaAs segments (as indicated in (a) by green boxes) with respect to d_0, for p = 1 (dark-blue squares) and 2 μm (orange circles), respectively. The region of the non-monotonic trend shown for both pitches (between d_0 = 30 to 70 nm) is marked with a red box – consistent behaviors with the results in the main manuscript.

REFERENCES

[1] Ruhstorfer, D.; Mejia, S.; Ramsteiner, M.; Döblinger, M.; Riedl, H.; Finley, J. J.; Koblmüller, G. Demonstration of n-type behavior in catalyst-free Si-doped GaAs nanowires grown by molecular beam epitaxy. *Appl. Phys. Lett.* **2020**, *116* (5), 052101.

[2] Ruhstorfer, D.; Lang, A.; Matich, S.; Döblinger, M.; Riedl, H.; Finley, J. J.; Koblmüller, G. Growth dynamics and compositional structure in periodic InAsSb nanowire arrays on Si (111) grown by selective area molecular beam epitaxy. *Nanotechnology* **2021**, *32* (13), 135604.

[3] Ajay, A.; Jeong, H.; Schreitmüller, T.; Döblinger, M.; Ruhstorfer, D.; Mukhundhan, N.; Koolen, P. A. L. M.; Finley, J. J.; Koblmüller, G. Enhanced growth and properties of non-catalytic GaAs nanowires via Sb surfactant effects. *Appl. Phys. Lett.* **2022**, *121* (7), 072107.

[4] Jeong, H. W.; Ajay, A.; Yu, H.; Döblinger, M.; Mukhundhan, N.; Finley, J. J.; Koblmüller, G. Sb-mediated tuning of growth- and exciton dynamics in entirely catalyst-free GaAsSb nanowires. *Small* **2023**, *19* (16), 2207531.

[5] Jeong, H. W.; Ajay, A.; Döblinger, M.; Sturm, S.; Gómez Ruiz, M.; Zell, R.; Mukhundhan, N.; Stelzner, D.; Lähnemann, J.; Müller-Caspary, K.; Finley, J. J.; Koblmüller, G. Axial growth characteristics of optically active InGaAs nanowire heterostructures for integrated nanophotonic devices. *ACS Appl. Nano Mater.* **2024**, *7* (3), 3032–3041.

[6] Rudolph, D.; Funk, S.; Döblinger, M.; Morkötter, S.; Hertenberger, S.; Schweickert, L.; Becker, J.; Matich, S.; Bichler, M.; Spirkoska, D.; Zardo, I.; Finley, J. J.; Abstreiter, G.; Koblmüller, G. Spontaneous alloy composition ordering in GaAs-AlGaAs core-shell nanowires. *Nano Lett.* **2013**, *13* (4), 1522–1527.

[7] Church, S. A.; Patel, N.; Al-Abri, R.; Al-Amairi, N.; Zhang, Y.; Liu, H.; Parkinson, P. Holistic nanowire laser characterization as a route to optimal design. *Adv. Opt. Mater.* **2023**, *11* (7), 2202476.

[8] Alanis, J. A.; Saxena, D.; Mokkapati, S.; Jiang, N.; Peng, K.; Tang, X.; Fu, L.; Tan, H. H.; Jagadish, C.; Parkinson, P. Large-scale statistics for threshold optimization of optically pumped nanowire lasers. *Nano Lett.* **2017**, *17* (8), 4860–4865.

[9] Heiss, M.; Conesa-Boj, S.; Ren, J.; Tseng, H.-H.; Gali, A.; Rudolph, A.; Uccelli, E.; Peiró, F.; Morante, J. R.; Schuh, D.; Reiger, E.; Kaxiras, E.; Arbiol, J.; Fontcuberta i Morral, A. Direct correlation of crystal structure and optical properties in wurtzite/zinc-blende GaAs nanowire heterostructures. *Phys. Rev. B* **2011**, *83* (4), 045303.

[10] Spirkoska, D.; Arbiol, J.; Gustafsson, A.; Conesa-Boj, S.; Glas, F.; Zardo, I.; Heigoldt, M.; Gass, M. H.; Bleloch, A. L.; Estrade, S.; Kaniber, M.; Rossler, J.; Peiro, F.; Morante, J. R.; Abstreiter, G.; Samuelson, L.; Fontcuberta i Morral, A. Structural and optical properties of high-quality zinc-blende/wurtzite GaAs nanowire heterostructures. *Phys. Rev. B* **2009**, *80* (24), 245325.

[11] Faria Junior, P. E.; Campos, T.; Bastos, C. M. O.; Gmitra, M.; Fabian, J.; Sipahi, G. M. Realistic multiband k·p approach from ab initio and spin-orbit coupling effects of InAs and InP in wurtzite phase. *Phys. Rev. B* **2016**, *93* (23), 235204.

[12] Panda, J. K.; Chakraborty, A.; Ercolani, D.; Gemmi, M.; Sorba, L.; Roy, A. Type II band alignment in InAs zinc-blende/wurtzite heterostructured nanowires. *Nanotechnology* **2016**, *27* (41), 415201.
